# PATIENT PORTAL USE IN FAMILY CAREGIVERS OF PATIENTS WITH DEMENTIA OR CANCER: THE NATIONAL STUDY OF CAREGIVING

**DOI:** 10.1093/geroni/igad104.3515

**Published:** 2023-12-21

**Authors:** Reed Bratches, Jaclyn Wall, Frank Puga, Giovanna Pilonieta, Marie Bakitas, David Geldmacher, J Nicholas Odom

**Affiliations:** The University of Alabama at Birmingham, Birmingham, Alabama, United States; The University of Alabama at Birmingham, Birmingham, Alabama, United States; The University of Alabama at Birmingham, Birmingham, Alabama, United States; The University of Alabama at Birmingham, Birmingham, Alabama, United States; The University of Alabama at Birmingham, Birmingham, Alabama, United States; The University of Alabama at Birmingham, Birmingham, Alabama, United States; The University of Alabama at Birmingham, Birmingham, Alabama, United States

## Abstract

Family caregivers are often inexperienced and require information from clinic visits to effectively provide care for patients. The patient portal is a common way for health systems to engage family caregivers, especially those of patients with dementia or cancer. The objective of our study was to analyze a large, nationally representative sample of family caregivers from the National Study of Caregiving (NSOC) to determine factors associated with patient portal use among family caregivers of persons with dementia and of persons with cancer. We conducted a secondary data analysis using data from the 2020 NSOC, a large nationally-representative survey of family caregivers. Weighted regression analysis was used to examine associations between family caregiver use of the patient portal and demographic variables including age, race/ethnicity, gender, employment status, caregiver health, education, and religiosity. A total of 462 participants (4,589,844 weighted) were analyzed. In the fully adjusted regression model for caregivers of persons living with dementia, Hispanic ethnicity was associated with a higher odds of patient portal use (OR: 2.81; 95%CI 1.05, 7.57; p=0.04) and having less than a college degree was associated with a lower odds of patient portal use (OR 0.36; 95%CI 0.18, 0.71; p=< 0.001). In the fully adjusted regression model for caregivers of persons living with cancer, no variables were associated with patient portal use. As the patient portal is a common method of connecting caregivers with information from clinic visits, future research should focus on understanding how the portal is used by the groups we have identified, and why.

